# Predictable Chikungunya Infection Dynamics in Brazil

**DOI:** 10.3390/v14091889

**Published:** 2022-08-26

**Authors:** Laith Yakob

**Affiliations:** Department of Disease Control, Faculty of Infectious & Tropical Diseases, London School of Hygiene & Tropical Medicine, London WC1H 9SH, UK; laith.yakob@lshtm.ac.uk; Tel.: +44-0207-927-2684

**Keywords:** arbovirus, epidemiology, Aedes, intervention, transmission

## Abstract

Chikungunya virus (CHIKV) was first imported into the Caribbean in 2013 and subsequently spread across the Americas. It has infected millions in the region and Brazil has become the hub of ongoing transmission. Using Seasonal Autoregressive Integrated Moving Average (SARIMA) models trained and validated on Brazilian data from the Ministry of Health’s notifiable diseases information system, we tested the hypothesis that transmission in Brazil had transitioned from sporadic and explosive to become more predictable. Consistency weighted, population standardized kernel density estimates were used to identify municipalities with the most consistent inter-annual transmission rates. Spatial clustering was assessed per calendar month for 2017–2021 inclusive using Moran’s I. SARIMA models were validated on 2020–2021 data and forecasted 106,162 (95%CI 27,303–200,917) serologically confirmed cases and 339,907 (95%CI 35,780–1035,449) total notifications for 2022–2023 inclusive, with >90% of cases in the Northeast and Southeast regions. Comparing forecasts for the first five months of 2022 to the most up-to-date ECDC report (published 2 June 2022) showed remarkable accuracy: the models predicted 92,739 (95%CI 20,685–195,191) case notifications during which the ECDC reported 92,349 case notifications. Hotspots of consistent transmission were identified in the states of Para and Tocantins (North region); Rio Grande do Norte, Paraiba and Pernambuco (Northeast region); and Rio de Janeiro and eastern Minas Gerais (Southeast region). Significant spatial clustering peaked during late summer/early autumn. This analysis highlights how CHIKV transmission in Brazil has transitioned, making it more predictable and thus enabling improved control targeting and site selection for trialing interventions.

## 1. Introduction

Chikungunya virus (CHIKV) is an enveloped, single-stranded RNA virus that belongs to the Alphavirus genus, Togaviridae family [1]. It is transmitted by the bite of an infectious mosquito, usually *Aedes aegypti* or *Ae*. *albopictus*. Most infections are symptomatic and symptoms during the acute phase include rash, high fever, headache and arthralgia [2]. Death can occur through multiple organ dysfunction syndrome during the acute (3 weeks) or sub-acute (3 weeks to 3 months) phase [3]. Chronic infection usually comprises of arthralgia lasting over 3 months, often for over a year.

Three major CHIKV genotypes have been identified: West African, East/Central/South African (ECSA) and Asian [4]. In 2004, a major epidemic of ECSA genotype CHIKV started in coastal Kenya, spreading to several Indian Ocean Islands, India, and South East Asia, infecting millions [5]. In December 2013, the Asian genotype was identified in the Caribbean and subsequently spread across the Americas [6]. The ECSA genotype was newly identified in Feira de Santana, Brazil, 6 months later and it is believed that this genotype is responsible for most ongoing Brazilian transmission [7].

An immunologically naïve population coupled with high densities of competent vectors resulted in 659,367 cases reported from the Americas by September 2014. In response, the Defense Advanced Research Projects Agency launched a chikungunya challenge to solicit forecasts for the major epidemic. The challenge was to predict transmission dynamics (e.g., week of peak incidence) over a 6-month period to inform public health responses. Several competitors were successful in producing accurate predictions using standard methods for assessing outbreaks, including mathematical models and statistical approaches for fitting cumulative incidence to growth curves [8].

It appears though the epidemiology of CHIKV has since transitioned; circulation of the virus in Brazil is now characterized by endemic transmission. By definition, this should mean CHIKV transmission has become more predictable in Brazil. This study sought to test this hypothesis and in doing so improve understanding of the contemporary epidemiology of CHIKV in Brazil. CHIKV data from the national notifiable disease information system were analyzed to determine whether incidence could be predicted using time series data forecasting models. The goal was to produce accurate predictions with sufficient lead-time to actually enable adaptive governmental responses to enhance public health interventions. A secondary goal was to identify priority areas within Brazil that had consistent high-level transmission to help site selection for trialing interventions.

## 2. Materials and Methods

**Figure 1:**
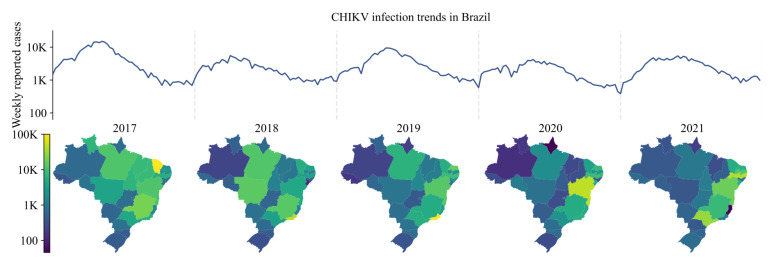
Weekly notifications of CHIKV infection 2017–2021, Brazil (**top**). State-level annual notifications of CHIKV infection 2017–2021 (**bottom**). Please note log scale.

All CHIKV infection data were anonymized and reported at the municipality level. These data were obtained from the Brazilian Ministry of Health’s notifiable diseases information system’s online SINAN (Sistema de Informação de Agravos de Notificação) database which distinguishes lab-confirmed (ELISA) from clinically diagnosed cases [9]. Respectively, 12%, 16%, 21%, 41% and 29% of cases were confirmed by serology for years 2017–2021. Figure 1 shows the trends in reported cases per Brazilian state for the period 2017–2021.

Seasonal Autoregressive Integrated Moving Average (SARIMA) models were trained on monthly cases from January 2017 to December 2019 and then validated on 2020 and 2021 data before forecasting for 2022–2023. SARIMA models are extensions of ARIMA models–one of the most widely used forecasting methods for univariate time series data forecasting. However, SARIMA models additionally allow for the direct modelling of the seasonal component of time series–a necessary feature for accommodating the pronounced seasonality of arbovirus infections in Brazil [10].

SARIMA models are of the form: (*p*, *d*, *q*) (*P*, *D*, *Q*); where *p* is the order of the autoregressive component, *d* is the order of the differencing, *q* is the order of the moving average component, *P* is the order of the seasonal autoregressive component, *D* is the order of the seasonal differencing, and *Q* is the order of the seasonal moving average component [11]. The general form of the model is:

(1) $$\phi_{p}\left(B\right)\Phi \left(P\right)\left(B^{s}\right){\left(1-B\right)}^{d}{\left(1-B^{s}\right)}^{D}Y_{t}=\theta_{q}\left(B\right)\Theta_{Q}\left(B^{s}\right)a_{t}$$

where *B* represents the backward shift operator, *s* is the known number of seasons per timeframe, *Y_t_* is the autoregressive moving average, *a_t_* is the unknown random error at time *t*. The ϕ and θ are, respectively, the autoregressive parameters and the moving average parameters to be estimated.

**Figure 2:**
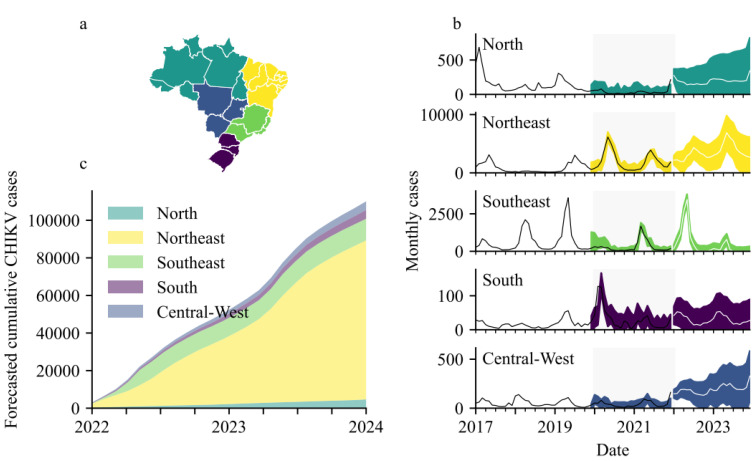
(**a**) How the 26 Brazilian states are categorized according to Region. (**b**) Region level SARIMA models with the grey segments denoting model validation time window (please note different scale on y-axes). (**c**) Forecasted cumulative CHIKV cases (serologically confirmed).

Parametrization required fitting data to alternative SARIMA models using the modified Powel method [12] and selecting the model with lowest Akaike Information Criterion. SARIMA models were assumed to have a seasonality lag of 12 time steps (i.e., seasonality repeats every 12 months) and were trained and validated separately for each Brazilian region (*n* = 5, see Figure 2a).

As of June 2022, the SINAN database had very limited information on CHIKV notifications for 2022 so these were excluded from the SARIMA model fitting and validation. To ascertain whether the forecasts for the first 5 months of 2022 (beginning of January to end of May) were on track, we compared our forecasts to data obtained for CHIKV notifications from the European Centre for Disease Prevention and Control (ECDC).

Next, Brazilian municipalities (*n* = 5570) were filtered with those that had infections consistently every year since 2017 being retained. A kernel density plot was generated for these areas of consistent transmission and weighted according to the minimum annual rate of infection standardized to local municipality population. The latest census was over 10 years ago. Instead we used 2020 estimates produced by the Brazilian Institute of Geography and Statistics [13]. The general equation for kernel density estimation is:

(2) $$f\left(x|h\right)\frac{1}{n}\overset{n}{\underset{i=1}{\sum }}\frac{1}{h}K\left(\frac{x-X_{i}}{h}\right)$$

where *f* is the kernel density estimate for the probability density function, *X* = {*X_1_*,…, *X_n_*} is a sample of *n* points drawn from the density function, *K* is the kernel and *h* is the smoothing bandwidth.

Evidence for significant disease clustering was sought by calculating Moran’s I statistic for population standardized infections per calendar month at the Brazilian state level. Moran’s I is defined as [14]:

(3) $$I=\frac{N}{W}\frac{\Sigma_{i}\Sigma_{j}w_{ij}\left(x_{i}-\bar{x}\right)\left(x_{j}-\bar{x}\right)}{\Sigma_{i}{\left(x_{i}-\bar{x}\right)}^{2}}$$

where *N* is the spatial unit number with indices *i* and *j*, *W* is the sum of all elements of a matrix of spatial weights (*w_ij_*) and $\bar{x}$ is the mean of the variable of interest x. This statistic was estimated computationally using a permutation approach [15]. A reference distribution for the statistic was calculated under the null hypothesis of spatial randomness by randomly permuting the observed values over the locations and computing Moran’s I. This resulted in a reference distribution which could then be plotted and used to estimate a pseudo *p*-value. Owing to the high number of Brazilian municipalities with missing data, global clustering was estimated at the state level. Distrito Federal is contained entirely within the state of Goiás and so separate data on its cases and population were merged with Goiás.

## 3. Results

Diagnostic plots for the SARIMA models comprise the standardized residuals, normal Q-Q plot and the correlogram. Diagnostics of SARIMA models for serologically confirmed cases in the five regions all generally indicated adequate model fits (Figures S1–S5 in Supplementary Materials). Final SARIMA models are shown in Figure 2b and details of their specifications, including their root mean squared errors, are in the (Supplementary Materials Table S1). Over the two years 2022–2023, the following case numbers were forecasted:- North: 4672 (95%CI 173–13,773); Northeast: 84,625 (95%CI 19,389–153,832); Southeast: 11,519 (95%CI 7141–21,963); South: 727 (95%CI 16–2178); and, Central-West: 4619 (95%CI 584–9171) (Figure 2c). The analysis was repeated to include all CHIKV notifications instead of only the lab-confirmed cases (Figure S6). For all reported cases, SARIMA models forecasted the following over the two years 2022–2023:- North: 7366 (95%CI 0–47,214); Northeast: 179,071 (95%CI 0–579,331); Southeast: 142,011 (95%CI 35,262–338,305); South: 1623 (95%CI 518–3130); and, Central-West: 9836 (95%CI 0–67,469).

Comparing our SARIMA models, generated without using any 2022 data, to CHIKV notifications in the first five months of 2022 (using data from ECDC because the SINAN database had not been updated to cover this period by the time of analysis) demonstrated remarkable accuracy. SARIMA forecasts estimated 92,739 (95%CI 20,685–195,191; see Figure S6) case notifications and the ECDC reported 92,349 case notifications [16].

**Figure 3:**
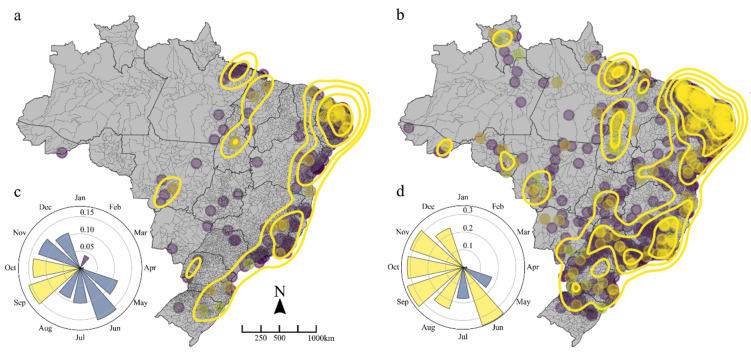
Municipalities consistently reporting serologically confirmed CHIKV infections (**a**), or all CHIKV notifications (**b**), every year 2017–2021 (points), overlaid with kernel density estimates weighted by the minimum annual rate of infection standardized to local municipality population. Contours were generated with bandwidth determined by Scott’s rule [17] adjusted by a factor of 0.4. Moran I estimates per calendar month show temporality of clustering for serologically confirmed (**c**) or all notification (**d**) CHIKV infections. Yellow months denote significant clustering (pseudo *p* value < 0.05) and blue months denote non-significant clustering (*p* ≥ 0.05).

Figure 3 shows the centroid locations of all municipalities that reported serologically confirmed (Figure 3a), and total notifications (Figure 3b) of, CHIKV infections after 2016. It also shows areas of sustained transmission over the five-year period in which at least 10 cases were reported per 100,000 population consistently each year. Overlaid on this are contours generated from kernel density estimates weighted by the minimum annual rate of infection standardized to local municipality population. These showed hotspots of consistent transmission in the states of Para and Tocantins (North region); Rio Grande do Norte, Paraiba and Pernambuco (Northeast region); and, Rio de Janeiro and eastern Minas Gerais (Southeast region). CHIKV notifications had similar distributions to serologically confirmed cases, however, with expanded areas of sustained transmission. These expanded areas additionally included Ceara and Piaui states in the Northeast, and eastern Sao Paolo in the Southeast.

Clustering was estimated using the global Moran’s I statistic. Using data of serologically confirmed cases only, a Moran’s I of 0.14 was estimated but the Monte Carlo simulation generated pseudo *p*-value was not significant at the 95% level (*p* = 0.078; see Figure S7 in Supplementary Materials for Moran scatter plot). When all CHIKV notifications were included (quadrupling the sample size), a Moran’s I of 0.28 was estimated and the Monte Carlo simulation generated pseudo *p*-value was 0.016 providing support for clustering at this level (see Figure S8). Clustering was then assessed for each calendar month (e.g., cases in January across all years 2017–2021 were combined = ‘Jan’) with the results shown for serologically confirmed cases, and all notifications, in Figure 3c,d, respectively. Monthly Moran’s I for serologically confirmed data showed significant clustering in September and October (Figure 3c), with the period of significant clustering extending from June to December when all notifications were analysed (Figure 3d).

## 4. Discussion

Over the past 20 years, chikungunya has emerged from relative obscurity to become an infection of widespread distribution and considerable global health importance [18]. By 2016, CHIKV infections were estimated to have incurred $185 billion in societal costs in the Americas [19]. Brazil has become the hub of CHIKV infections worldwide, with high incidence reported annually [20]. While transmission is generally reported to be unpredictable [21], this is likely due to a bias in reports focusing on large-scale outbreaks with much more limited information available on endemic settings [22]. Using five years of data (2017–2022), we sought to elucidate whether CHIKV transmission dynamics in Brazil had transitioned to become less erratic, and hence more predictable.

For each of the five regions, SARIMA models were trained on data from 2017–2019 prior to being validated on 2020–2021 data and then forecasted for 2022–2023. Validation diagnostics were good for models of all regions. The Northeast and Southeast have been worst affected (in terms of total case numbers) and were projected to continue bearing the brunt (>90%) of Brazilian infections. Combined, 106,162 (95%CI 27,303–200,917) serologically confirmed cases, and 339,907 (95%CI 35,784–1035,449) total notifications, were forecasted for the two years beginning January 2021. SARIMA models for 2022 up until June forecasted 92,739 (95%CI 20,685–195,191) total notifications and these closely matched a report from the ECDC, published on 2 June 2022, stating that 92,349 total notifications had been recorded in Brazil so far in 2022 [16].

SARIMA models have a track record in accurately forecasting infectious diseases. For example, US government agencies within the Pandemic Prediction and Forecasting Science and Technology Working Group launched an open dengue forecasting challenge in 2015, and SARIMA models generally performed well compared with more complex models, including having the best overall calibration and the highest skill forecasts for peak week [23]. One limitation of this forecasting method is when there is a paucity of data with which to train the model, e.g., early during an outbreak, SARIMA forecasts are considered accurate only for short time horizons (days-to-weeks). However, when data are sufficient for more prolonged model training (>3 years for seasonally cycling transmission), the utility of SARIMA forecasts can extend to months-to-years. Recent SARIMA models of Zika virus in Brazil also showed good predictive accuracy whereby 4643 (95%CI 309–19,831) confirmed cases were forecasted for 2021 [24], and SINAN reported 4092 confirmed cases for that year [9].

Accurate and timely forecasts for CHIKV infections can guide decision-making on medical countermeasures, allowing them to be used in more effective ways. Specifically, they enable anticipating resource requirements, refining situational awareness and monitoring control efforts [25]. Complementing our forecasts with maps of standardized CHIKV cases weighted by inter-annual transmission consistency had two purposes. First, it allowed for the spatial targeting of (vector management) interventions to prioritize the worst affected populations. Second, identifying areas of consistent transmission is of particular value to inform site selection for seroprevalence studies and intervention trials. For example, several CHIKV vaccine candidates are undergoing clinical trials [26]; but, because transmission is typically sporadic, a major hurdle for demonstrating efficacy at a phase III trial for an arbovirus vaccine is identifying which population(s) to enroll [27].

The biggest limitation of the current analysis pertains to the data. Laboratory testing is technically demanding and requires equipment that is not widely available in Brazil. As a consequence, barely a quarter of cases were serologically tested [28]. We tried to reconcile this disparity as well as we could by repeating all analyses and presenting results for serologically confirmed, as well as total case notifications. Further, acute symptoms are shared with several other infections, most notably dengue, easily resulting in clinical misdiagnosis. Brazil’s longer history with dengue, coupled with typically much higher dengue case numbers, means CHIKV infection misdiagnosis as dengue will tend to dominate the opposite scenario [29]. This will have the inevitable result of biasing notifications (and our forecasts) towards underestimation. One approach to counter underestimation would be to rescale reported cases to account for asymptomatic infections. However, there is worrying ambiguity over the proportional representation of sub-clinical CHIKV infection, with estimates ranging from 3–49% [30], so a singular scaling factor to inflate reported case numbers does not seem appropriate.

CHIKV infections incur over 100,000 Disability Adjusted Life Years per year, with most of the burden suffered in Brazil [31]. The forecasts generated in the current analysis have demonstrable accuracy and provide sufficient lead-time for guiding decision-making on medical countermeasures. This analysis also highlights how CHIKV transmission in this setting has transitioned, making it more predictable and thus enabling site selection for trialing interventions.

## Data Availability

All data are anonymized and are available from the Brazilian Ministry of Health’s notifiable diseases information system’s online SINAN database.

## Supplementary Materials

The following supporting information can be downloaded at: https://www.mdpi.com/article/10.3390/v14091889/s1. Figure S1: Diagnostic plots for SARIMA model for the North region of Brazil; Figure S2: Diagnostic plots for SARIMA model for the Northeast region of Brazil; Figure S3: Diagnostic plots for SARIMA model for the Southeast region of Brazil; Figure S4: Diagnostic plots for SARIMA model for the South region of Brazil; Figure S5: Diagnostic plots for SARIMA model for the Central-West region of Brazil; Figure S6: a) How the 24 Brazilian states are categorized according to Region. b) Region level SARIMA models with the grey segments denoting model validation time window. c) Forecasted cumulative CHIKV cases (all notifications); Figure S7: The estimated Moran’s I (orange) relative to the reference distribution generated through Monte-Carlo simulation (left); and, the associated Moran scatterplot (right). Results are for serologically confirmed CHIKV cases only; Figure S8: The estimated Moran’s I (orange) relative to the reference distribution generated through Monte-Carlo simulation (left); and, the associated Moran scatterplot (right). Results are for all CHIKV case notifications; Table S1: The specifications and random mean squared errors (RMSE) for the region-level SARIMA models.
