# Supplementary material for: Predictable Chikungunya Infection Dynamics in Brazil
Source: Viruses. 2022 Aug 26;14(9):1889. doi: 10.3390/v14091889 (PMC9505030; doi:10.3390/v14091889)
Supplement: Supplementary file 1 [file viruses-14-01889-s001.zip › viruses-1846341-supplementary.pdf]

Supplementary material for:

Predictable Chikungunya Infection Dynamics in Brazil

Laith Yakob

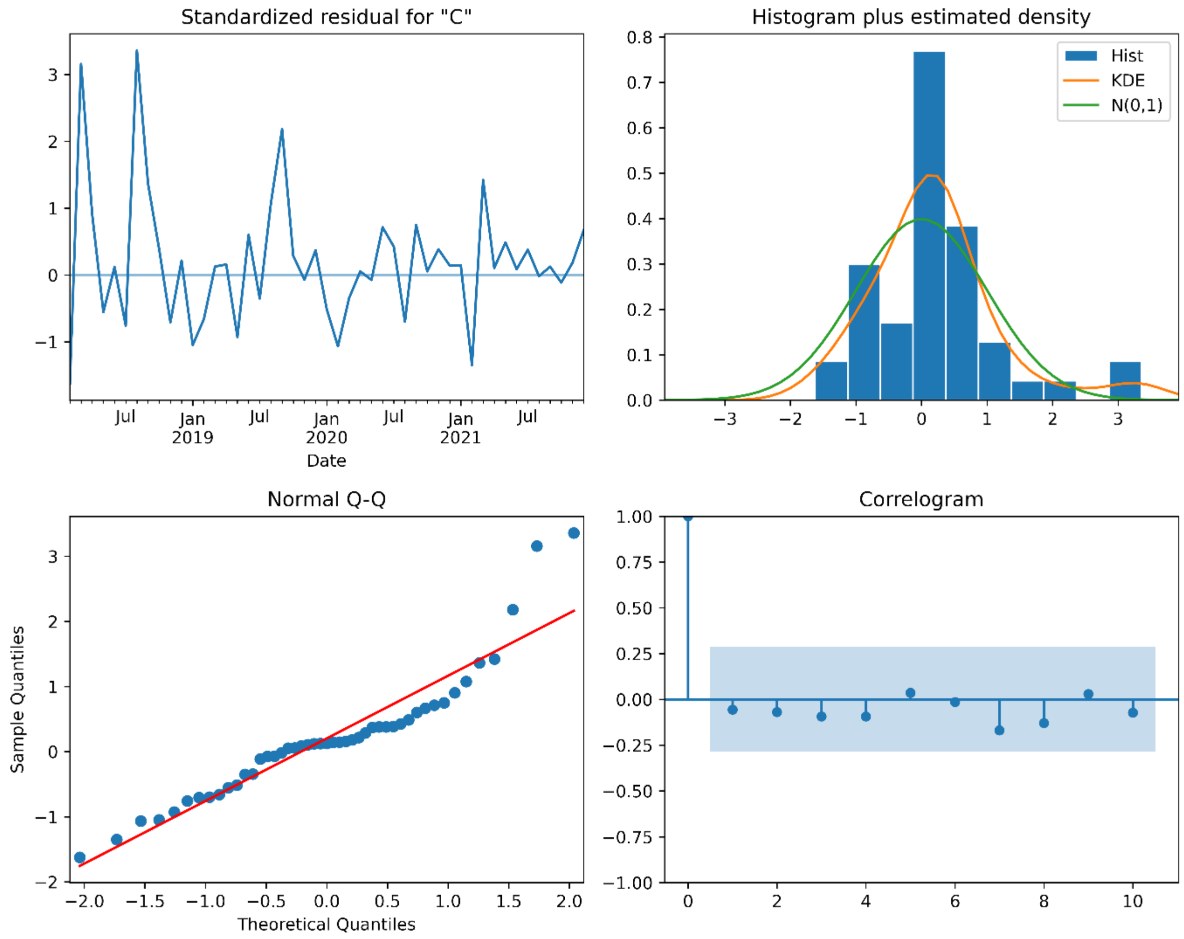

Figure S1. Diagnostic plots for SARIMA model for the North region of Brazil.

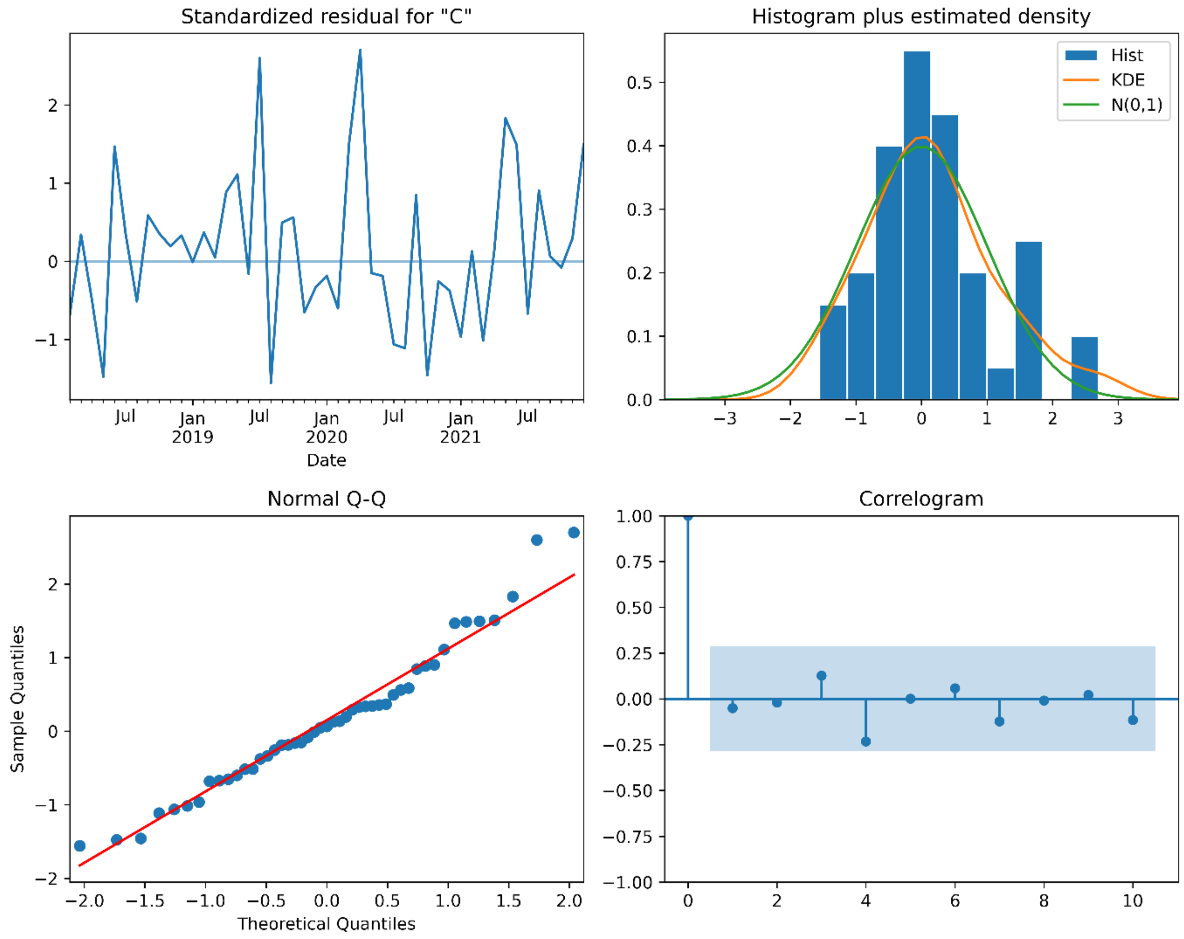

Figure S2. Diagnostic plots for SARIMA model for the Northeast region of Brazil.

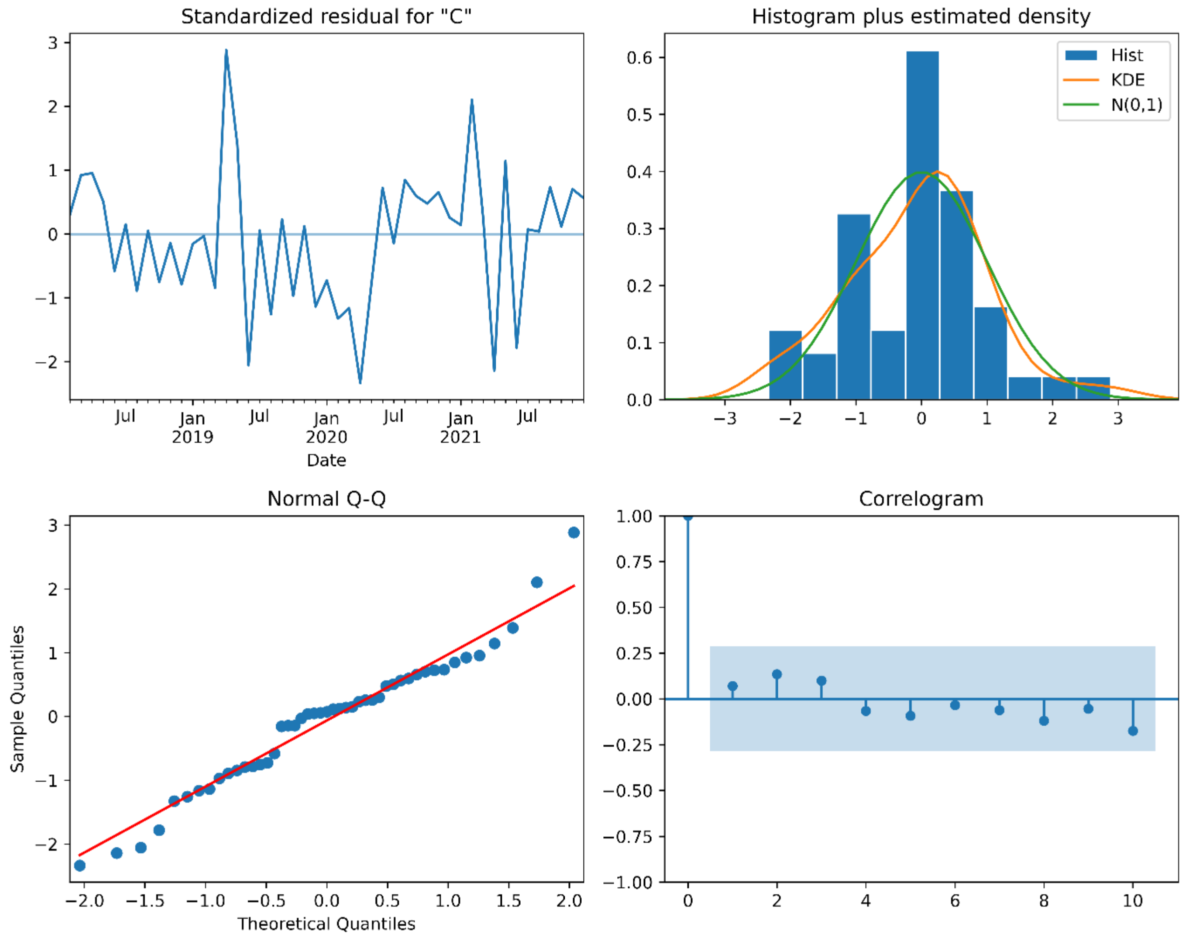

Figure S3. Diagnostic plots for SARIMA model for the Southeast region of Brazil.

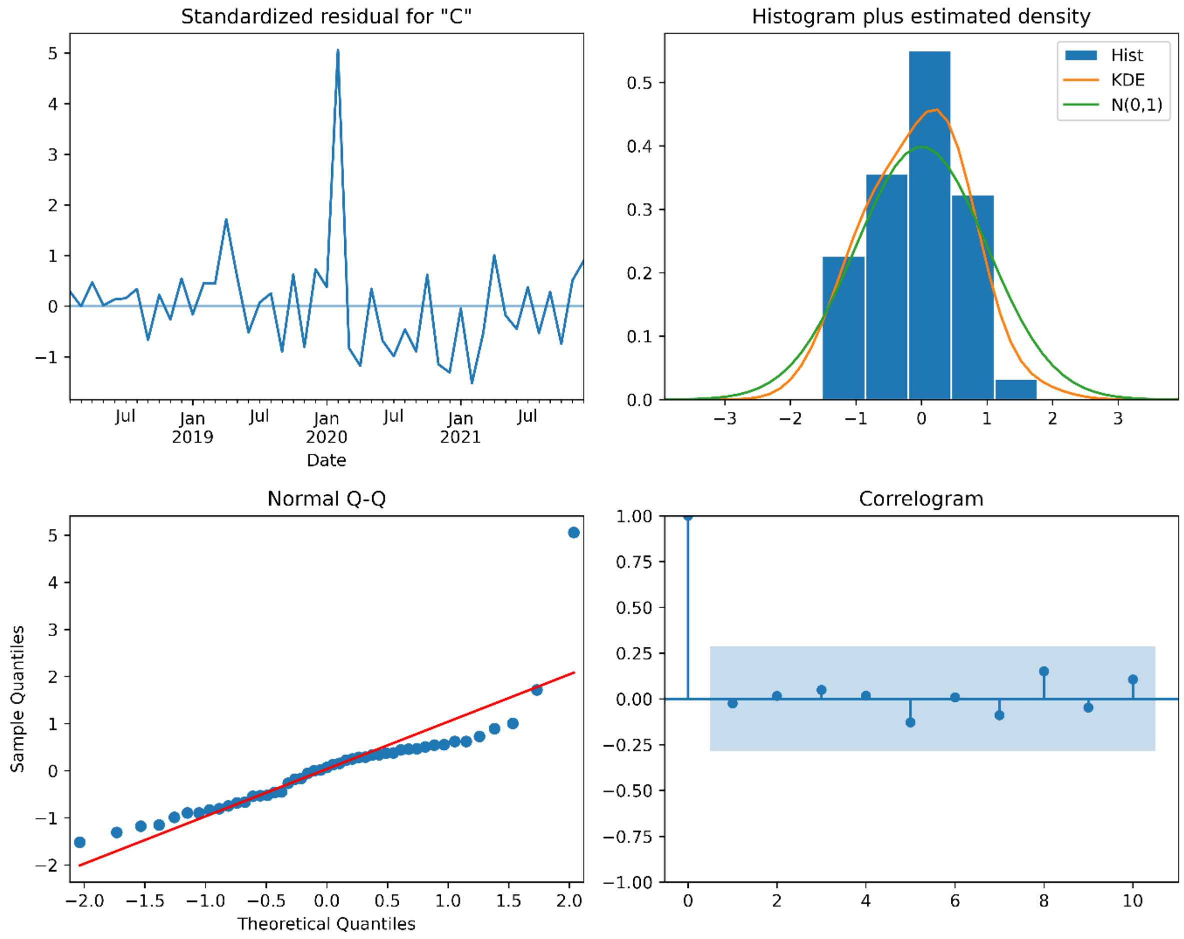

Figure S4. Diagnostic plots for SARIMA model for the South region of Brazil.

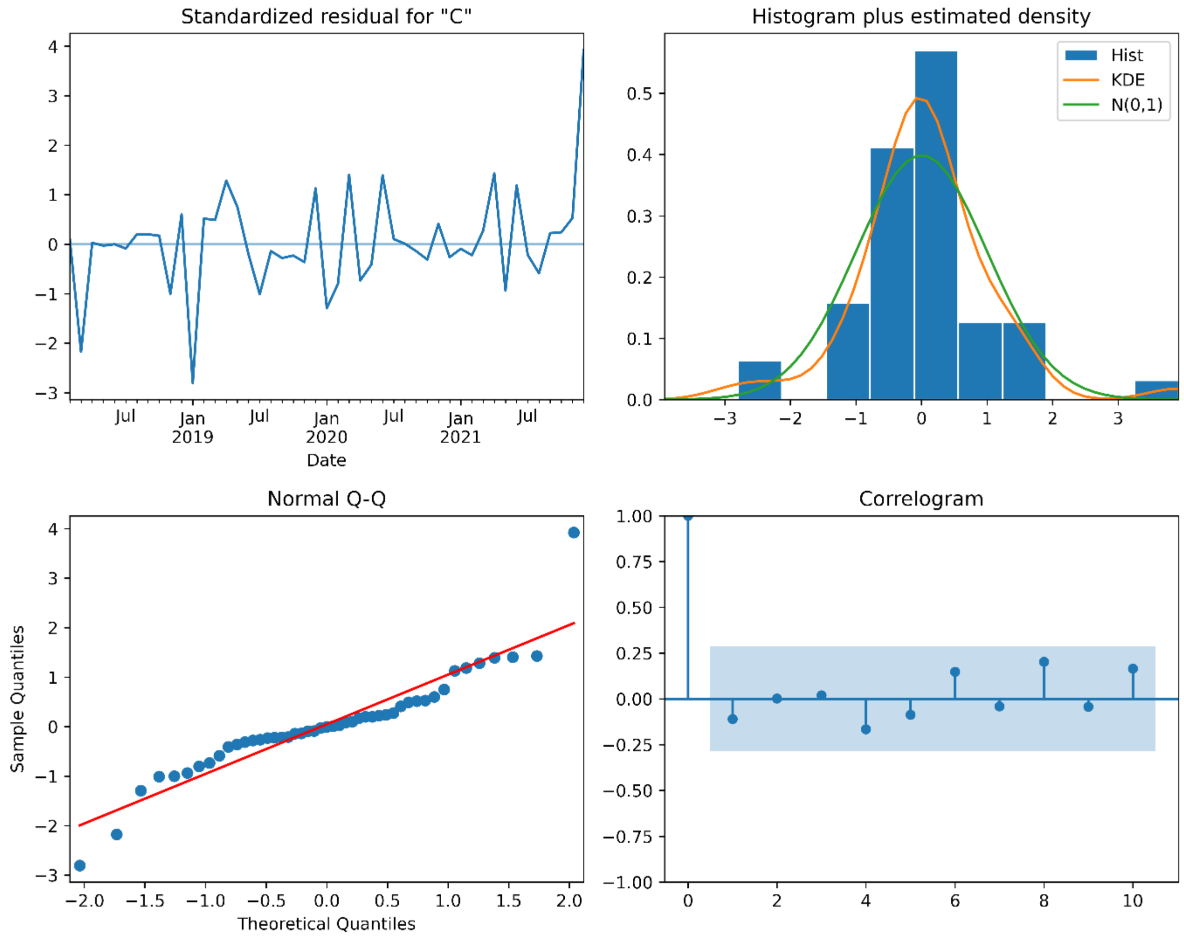

Figure S5. Diagnostic plots for SARIMA model for the Central-West region of Brazil.

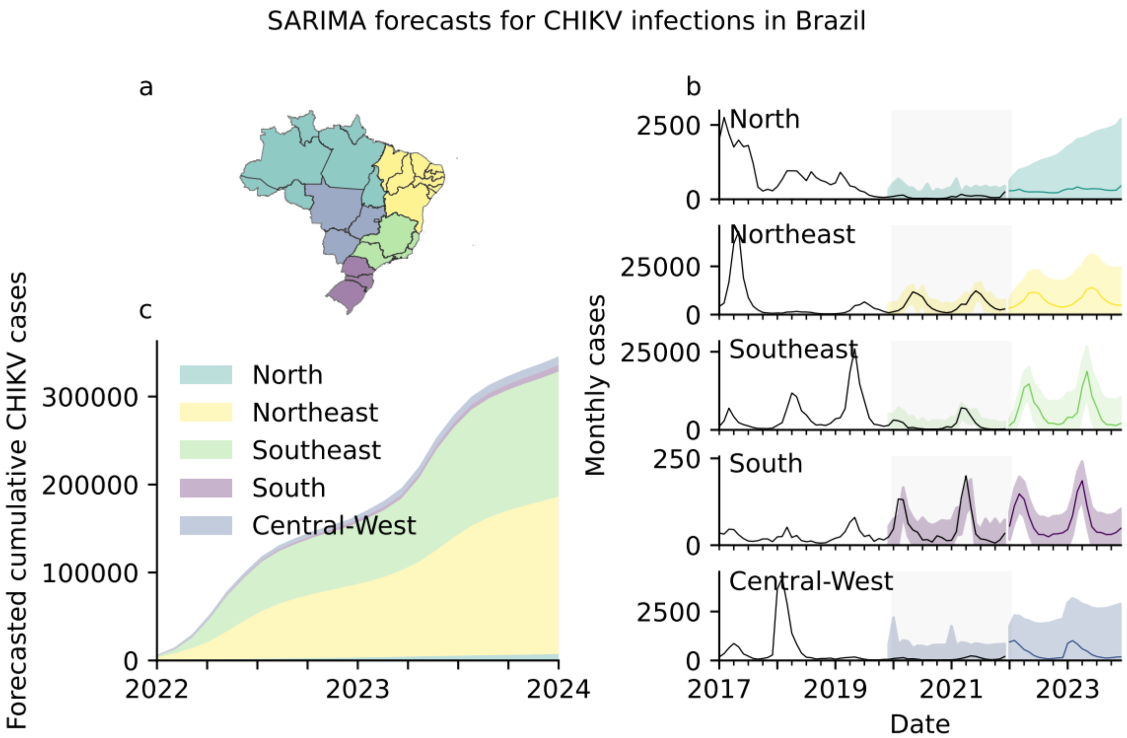

Figure S6. (**a**) How the 24 Brazilian states are categorized according to Region. (**b**) Region level SARIMA models with the grey segments denoting model validation time window. (**c**) Forecasted cumulative CHIKV cases (all notifications).

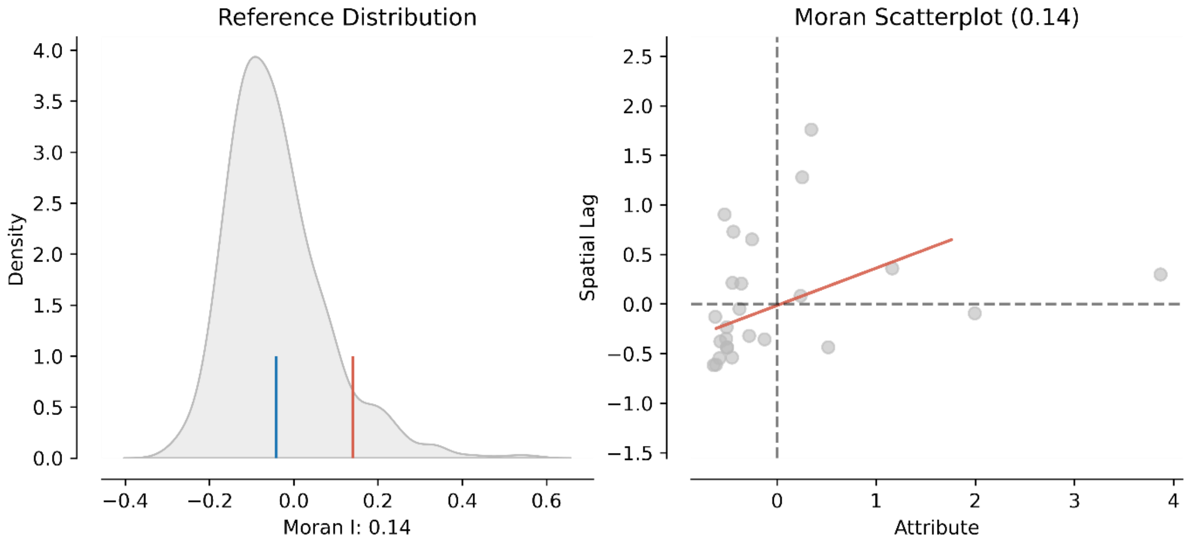

Figure S7. The estimated Moran's I (orange) relative to the reference distribution generated through Monte-Carlo simulation (left); and, the associated Moran scatterplot (right). Results are for serologically confirmed CHIKV cases only.

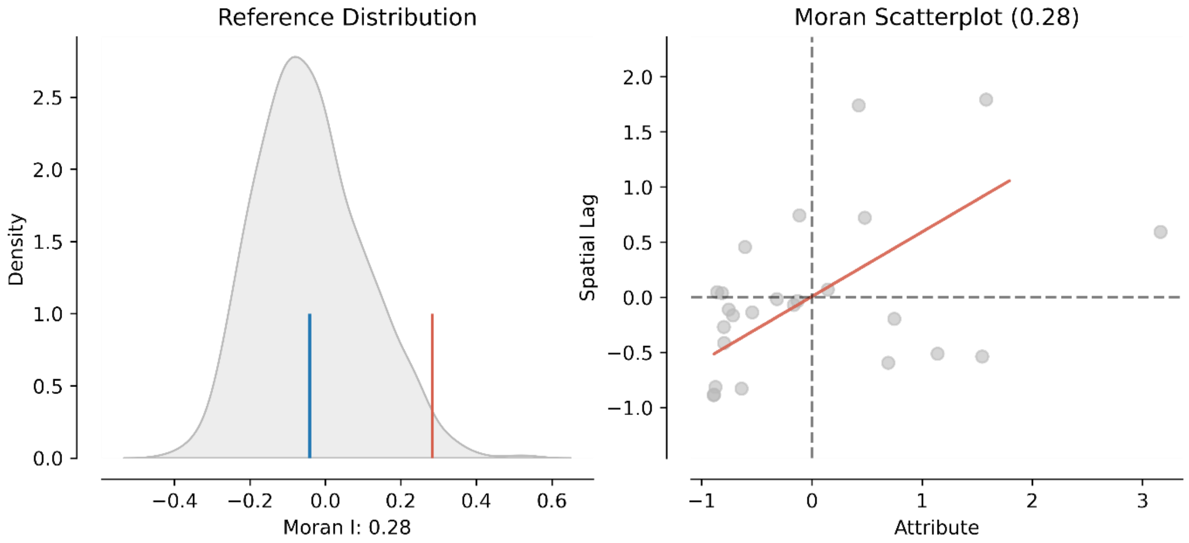

Figure S8. The estimated Moran's I (orange) relative to the reference distribution generated through Monte-Carlo simulation (left); and, the associated Moran scatterplot (right). Results are for all CHIKV case notifications.

Table S1. The specifications and random mean squared errors (RMSE) for the region-level SARIMA models

Region	Parameters for serologically confirmed SARIMA models	RMSE	Parameters for all-notification SARIMA models	RMSE
North	(1,1,3)(1,1,0)	43.49	(1,1,1)(1,1,0)	119.36
Northeast	(2,1,1)(2,1,0)	494.71	(0,1,3)(2,1,0)	1681.41
Southeast	(0,1,2)(2,1,0)	203.37	(2,1,0)(2,1,3)	894.19
South	(0,1,3)(0,1,1)	21.38	(2,1,1)(1,1,0)	30.47
Central-West	(0,1,0)(0,1,2)	29.56	(0,1,3)(0,1,1)	329.53
